# Collagen V insufficiency in a mouse model for Ehlers Danlos-syndrome affects viscoelastic biomechanical properties explaining thin and brittle corneas

**DOI:** 10.1038/s41598-021-96775-w

**Published:** 2021-08-30

**Authors:** Sabine Kling, Emilio A. Torres-Netto, Hormoz Abdshahzadeh, Edgar M. Espana, Farhad Hafezi

**Affiliations:** 1 OPTIC-Team, Computer Vision Laboratory, Department of Information Technology and Electrical Engineering, ETH Zurich, Sternwartstrasse 7, 8092 Zurich, Switzerland; 2 Laboratory of Ocular Cell Biology, CABMM, University of Zurich, Zurich, Switzerland; 3 ELZA Institute AG, Dietikon/Zurich, Switzerland; 4 Department of Ophthalmology, Paulista School of Medicine, Federal University of Sao Paulo, São Paulo, Brazil; 5 Faculty of Medicine, University of Geneva, Geneva, Switzerland; 6 Department of Ophthalmology, University Hospital Zurich, University of Zurich, Zurich, Switzerland; 7 Dept. of Ophthalmology, Morsani College of Medicine, University of South Florida, Tampa, FL USA; 8 Dept. of Ophthalmology, USC Roski Eye Institute, Keck School of Medicine, USC Los Angeles, Los Angeles, USA; 9 Dept. of Ophthalmology, Wenzhou Medical University, Wenzhou, China

**Keywords:** Experimental models of disease, Pathogenesis, Biomedical engineering

## Abstract

Ehlers–Danlos syndrome (EDS) is a genetic disease leading to abnormalities in mechanical properties of different tissues. Here we quantify corneal biomechanical properties in an adult classic EDS mouse model using two different measurement approaches suited for murine corneal mechanical characterization and relate differences to stromal structure using Second Harmonic Generation (SHG) microscopy. Quasi-static Optical Coherence Elastography (OCE) was conducted non-invasively during ambient pressure modulation by − 3 mmHg. 2D-extensometry measurements was conducted invasively consisting of a pre-conditioning cycle, a stress-relaxation test and a rupture test. In a total of 28 eyes from a *Col5a1*^+*/−*^ mouse model and wild-type C57BL/6 littermates (wt), *Col5a1*^+/−^ corneas were thinner when compared to wt, (125 ± 11 vs 148 ± 10 μm, respectively, p < 0.001). Short-term elastic modulus was significantly increased in OCE (506 ± 88 vs 430 ± 103 kPa, p = 0.023), and the same trend was observed in 2D-extensometry (30.7 ± 12.1 kPa vs 21.5 ± 5.7, p = 0.057). In contrast, in stress relaxation tests, *Col5a1*^+/−^ corneas experienced a stronger relaxation (55% vs 50%, p = 0.01). SHG microscopy showed differences in forward and backward scattered signal indicating abnormal collagen fibrils in *Col5a1*^+/−^ corneas. We propose that disturbed collagen fibril structure in *Col5a1*^+/−^ corneas affects the viscoelastic properties. Results presented here support clinical findings, in which thin corneas with global ultrastructural alterations maintain a normal corneal shape.

## Introduction

Ehlers–Danlos syndrome (EDS) is an autosomal dominant connective tissue disease primarily affecting skin and joints, and generally characterized by joint hypermobility, skin hyperextensibility and tissue fragility. Corneal thinning is a common finding in classic type EDS^1,2^, along with a steep cornea, but not a higher incidence of keratoconus^3^. On a molecular level, classic type EDS results from a mutation in COL5A1 and COL5A2.

Collagen V is present in the corneal stroma and makes approximately 10 to 20% of its collagen content, see review^4^. Collagen V is responsible for the formation of heterotypic fibrils together with collagen I^5,6^. The high content of collagen V in the cornea accounts for the unique small diameter of corneal fibrils^6,7^. *A Col5a1*^+/−^ mouse model of classic EDS, with a 49% reduced content of collagen V, was found to have a 26% thinner cornea and abnormalities in collagen fibrils morphology^2^.

Corneal biomechanics in patients with classic EDS is of translational relevance because of this apparent contradiction between significant corneal thinning and the normal incidence of corneal ectasia.

Here, we quantify, for the first time to our knowledge, the biomechanical properties of the cornea in a mouse model for classic type EDS, and compare two distinct measurement approaches.

## Results

### Optical coherence elastography (OCE)

**Figure 1:**
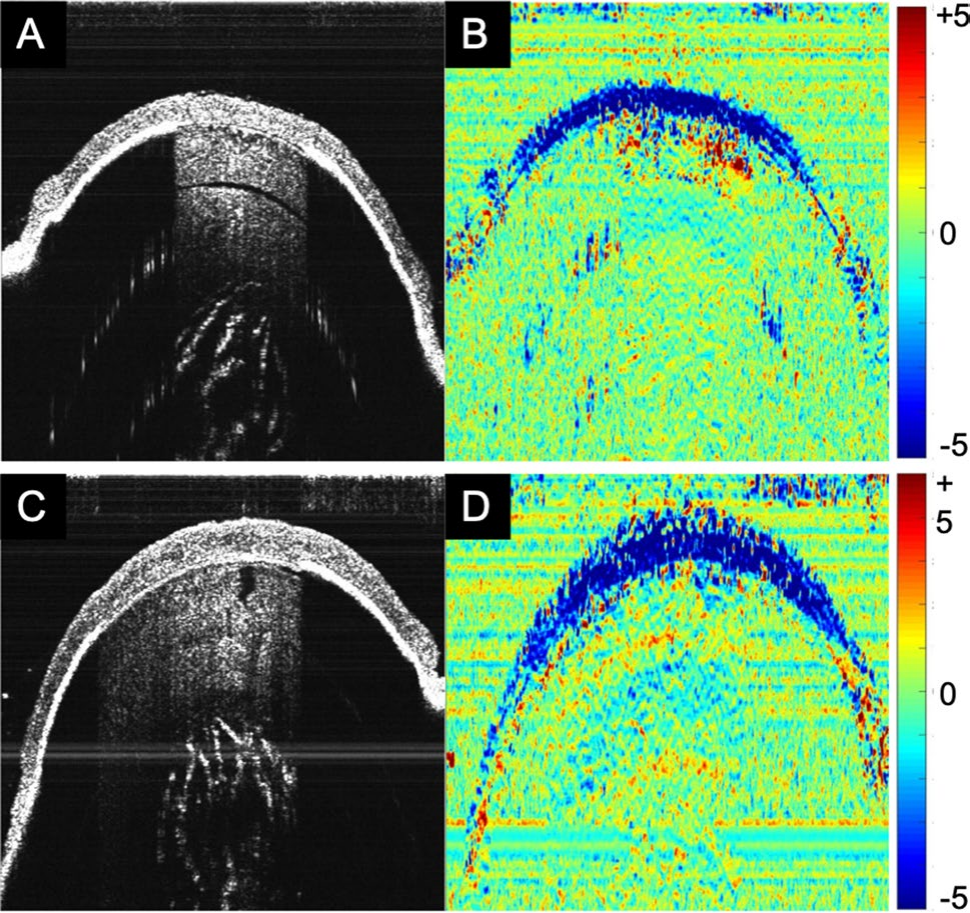
Structural image (**A**,**C**) and strain map (**B**,**D**) obtained from OCT. *Col5a1*^+/−^ eyes (**A**,**B**) had a thinner corneal thickness than wild-type eyes (**C**,**D**), but a similar axial strain distribution.

**Figure 2:**
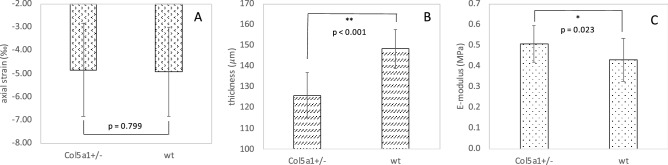
(**A**) Average induced axial corneal strain, (**B**) central corneal thickness and (**C**) E-modulus obtained from OCE in *Col5a1*^+/−^ eyes and wild-type eyes. * indicates statistical significance at p < 0.05, ** indicates statistical significance at p < 0.001. n = 14 per condition.

Optical coherence tomography was used for non-invasive structural and strain imaging. Figure 1 shows representative images in *Col5a1*^+/−^ and wt corneas. It is clearly visible that *Col5a1*^+/−^ corneas were thinner (125 ± 11 vs 148 ± 10 μm, factor 1.2) than wt corneas, with a significance of p < 0.001. Corneal strain images were relatively noisy, therefore for subsequent strain interpretation the mean value within a 21 × 21 pixels (68 × 63 μm) central area was computed. Figure 2A presents the corresponding mean axial strain values. No significant difference was found in axial strain (− 4.84 ± 1.99 vs − 4.93 ± 1.92‰, p = 0.799). After accounting for thickness (Fig. 2B), however, the E-modulus was significantly (p = 0.023) higher in the *Col5a1*^+/−^ corneas (506 ± 88 vs 430 ± 103 kPa, factor 1.2) indicating higher mechanical stiffness, see Fig. 2C.

### 2D extensometry

**Figure 3:**
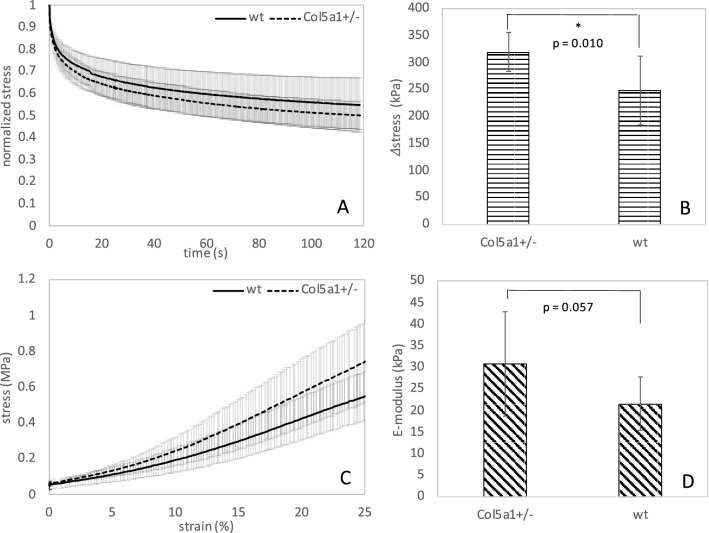
(**A**) Stress-relaxation curve (n = 12, *Col5a1*^+/−^ and n = 14, wt), (**B**) absolute stress reduction after relaxation (n = 12, *Col5a1*^+/−^ and n = 14, wt), (**C**) stress–strain curve (n = 8, Col5a1^+/−^ and n = 11, wt) and (**D**) average E-modulus between 10 and 15% of strain (n = 8, *Col5a1*^+/−^ and n = 11, wt) for wild-type and *Col5a1*^+/−^ corneas. * indicates statistical significance at p < 0.05.

Destructive extensometry is a standard technique for mechanical characterization. Stress-relaxation measurements could be interpreted in 12 out of 14 wt corneas and 9 out of 14 *Col5a1*^+/−^ corneas, and stress–strain measurements could be interpreted in 8 and 11 *Col5a1*^+/−^ and wt corneas, respectively, because of premature rupture of the sample during testing. Hence, *Col5a1*^+/−^ had an increased susceptibility to rupture by approx. 25–30%. This percentage represents the ratio of eyes that ruptured prematurely in extensometry tests versus eyes that could be completely tested. It should be noted that on average corneas ruptured at a load of 0.87 ± 24 N, with *Col5a1*^+/−^ showing a trend towards a slightly higher breaking load (p = 0.052). Figure 3A,B present results from the stress-relaxation analysis. *Col5a1*^+/−^corneas did present a significantly (p = 0.010) higher decrease in stress after 100 s of relaxation than wt corneas (55% vs 50%) indicating a stronger relaxation and thus more viscosity, i.e. less temporal stability. When fitting a 3-terms Prony series to the two relaxation curves, the following constants are obtained for wt corneas: E_∞_ = 1.18 MPa, E_1_ = 0.43 MPa, E_2_ = 0.51 MPa, E_3_ = 0.88 MPa, τ_1_ = 3.1 s, τ_2_ = 51 s, τ_3_ = 124 s. And for Col5a1^+/−^ corneas: E_∞_ = 1.14 MPa, E_1_ = 0.50 MPa, E_2_ = 0.62 MPa, E_3_ = 1.29 MPa, τ_1_ = 3.1 s, τ_2_ = 56 s, τ_3_ = 57 s. Figure 3C,D present results from the stress–strain analysis computed from the initial region of the rupture test. Interestingly, and similar to OCE measurements, *Col5a1*^+/−^corneas showed a trend towards a steeper stress–strain curve and thus higher E-modulus than wt corneas (30.7 ± 12.1 kPa vs 21.5 ± 5.7), even though statistical significance was not reached (p = 0.057).

### Second harmonic generation imaging

**Figure 4:**
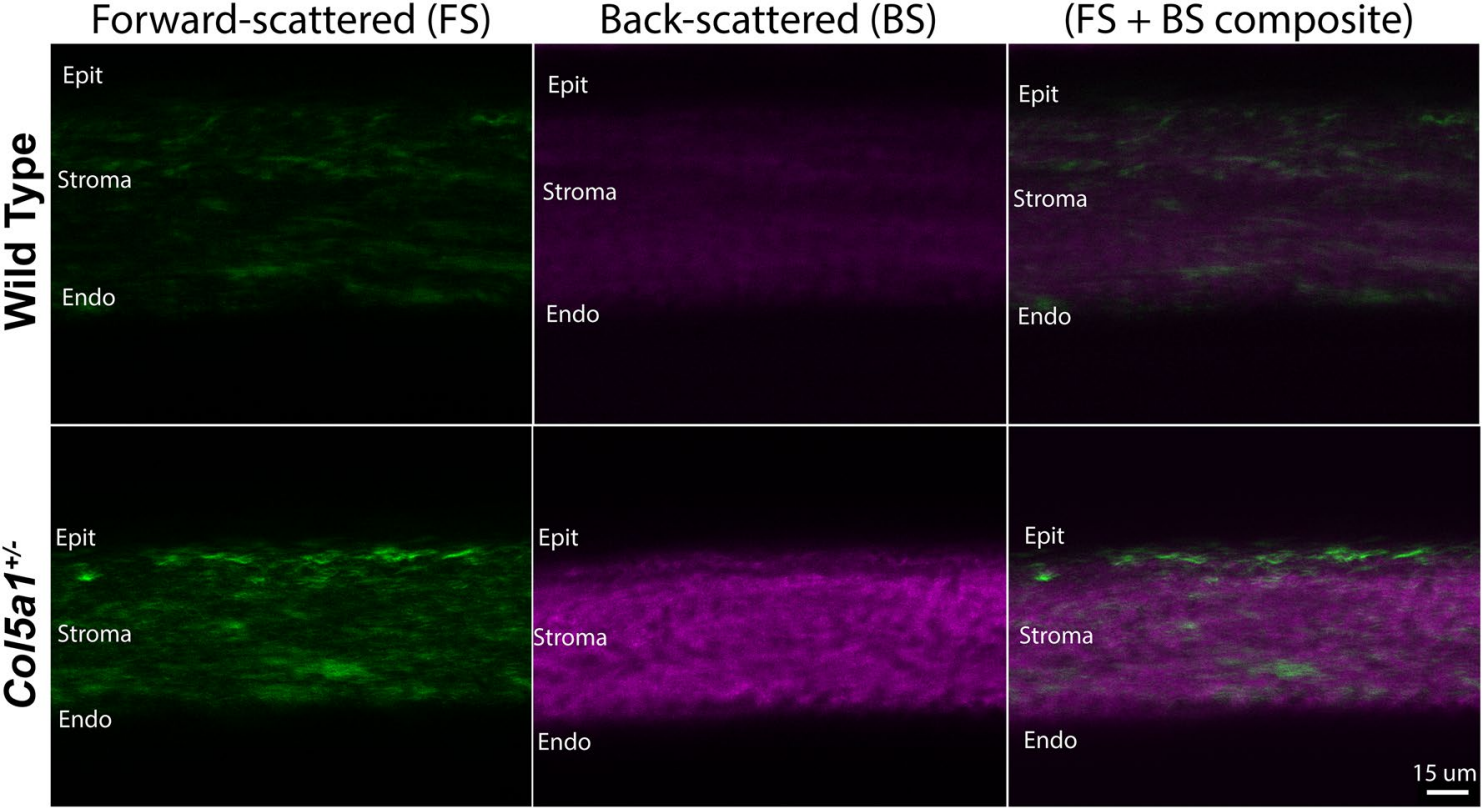
Cross-section SHG images of corneas obtained immediately after enucleation and without tissue fixation or manipulation. Weaker forward-scattered and backward-scattered signal is noted in the wt corneas. Representative images of *Col5a1*^+/−^ (n = 6) and wt (n = 6) corneas.

**Figure 5:**
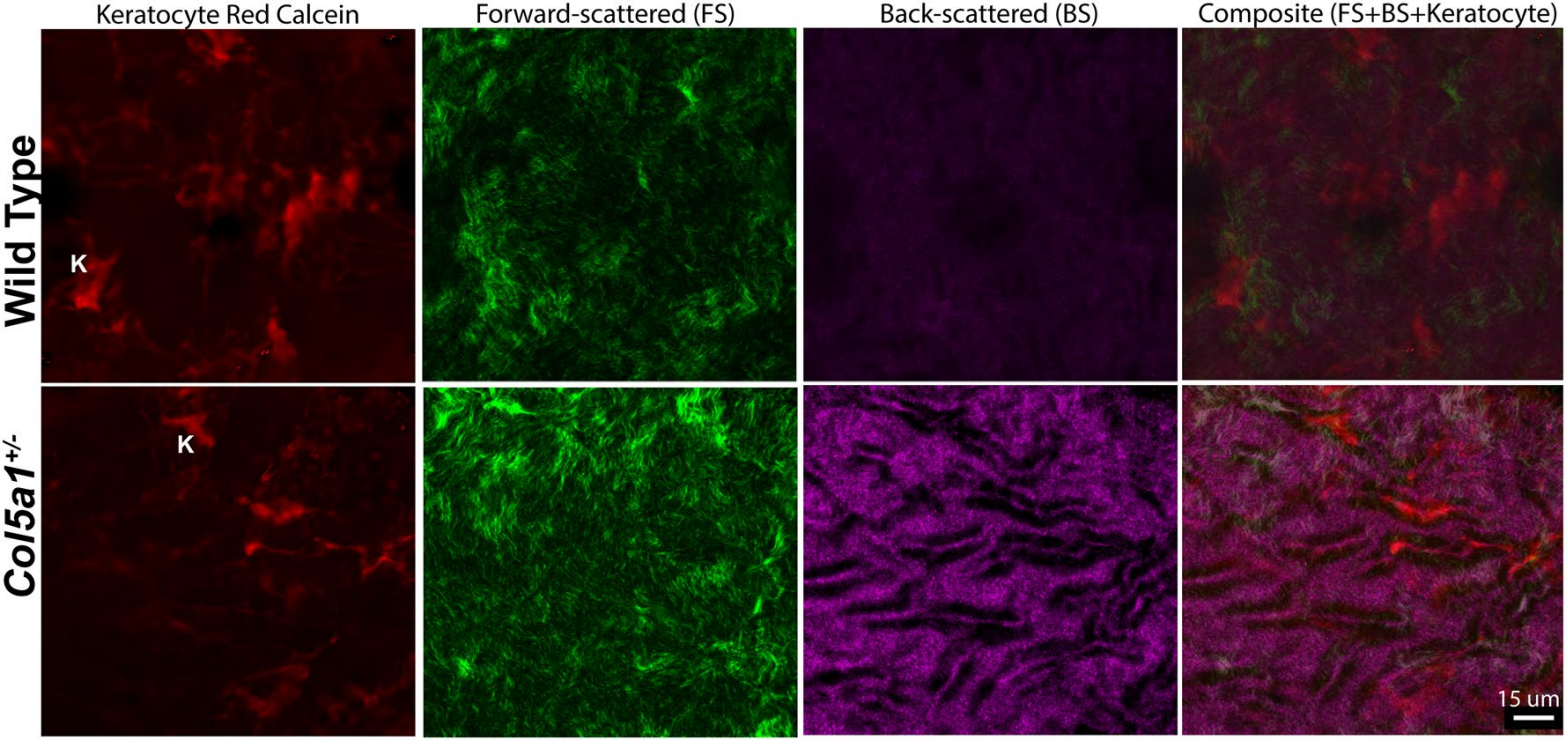
En face SHG images obtained after corneas were dissected from the globe immediately after enucleation and imaged. Keratocytes (K) are shown in red, as the live cell dye Calcein AM penetrates the live keratocyte cell membrane. Note the differences is keratocyte shape in *Col5a1*^+/−^ compared to wt and the increase in stromal folds noted in the *Col5a1*^+/−^ corneas obtained with back scattered signal. Representative images of *Col5a1*^+/−^ (n = 6) and wt (n = 6) corneas.

Second harmonic generation imaging was performed to evalute stromal hierarchical organization in a COL5A1 deficient matrix. To assess the influence of COL5A1 on fibrillar collagen and hierarchical organization without tissue manipulation or chemical fixation, we studied adult *Col5a1*^+/−^ stromas and compared them to WT stromas. En face imaging of the stroma and cross sections of adult corneas from both groups were imaged immediately after enucleation. Increased forward scattered and backward scattered signaling suggests fibril disorganization, increased fibril density and abnormalities in the hierarchical organization in *Col5a1*^+/−^ corneas compared to WT, see Figs. 4 and 5. These findings suggest that a matrix deficient in COL5A1 presents with changes in fibril structure and stromal hierarchical organization.

## Discussion

Our analyses confirm that *Col5a1*^+/−^ corneas represent a valid model for classic type EDS for the following reasons: first, an increased susceptibility to corneal rupture, about 25–30%, was observed. This is particularly interesting, as the remaining Col5a1^+/−^ showed the trend to a higher load necessary for tissue break and thus premature rupture cannot be attributed to the fact that corneal tissue was thinner in those corneas. Spontaneous tissue rupture is more typical for type VI EDS. In a clinical group of 11 patients^8^, corneal rupture occurred spontaneously or after minimal trauma in approximately 35%. This condition is also known as ‘oculus fragilis’^9^. Interestingly, classic type EDS has also been associated with spontaneous arterial rupture^10^. We may speculate that a mutation in collagen V leads to a similar structural defect created by a deficiency in lysyl hydroxylase, except that rupture occurs at higher loads and thus is less frequent. Mechanically, spontaneous rupture might be attributed to a stiffer short-term behavior (increased E-modulus, Figs. 2C, 3D), but potentially more brittle corneal tissue.

Second, a thinner cornea in *Col5a1*^+/−^ eyes of approximately 20% was found in the current study, which is in line with previous literature reporting 26% thickness reduction in the same mouse model^2^. Compared to a clinical group of EDS patients, this feature appears to manifest in humans less pronouncedly. Here, a mean reduction of 41 μm was reported^3^, which corresponds to a thickness reduction by approximately 8% assuming a normal corneal thickness of 546 μm^11^. Corneal thinning in *Col5a1*^+/−^ corneas might be attributed to a stronger stress relaxation under the same load as applied in this study (Fig. 3B), or correspondingly under normal IOP in patients.

While this combination—i.e. increased relaxation in combination with increased brittleness—may appear contradictory, it could be explained by a difference in time-scale: Stress relaxation was conducted during 120 s, while the rupture test took only 12 s (for the strain region analyzed). These measurement settings had been previously applied for the characterization of mouse corneas after UV riboflavin cross-linking^12,13^ and did allow a robust fit to Eq. (7) and to find meaningful differences. Similarly, the OCE measurement was conducted within < 20 s. This means time scales vary by a factor of 5 to 10. It is normal for viscoelastic material to behave stiffer in shorter time intervals^14^. In the context of this study, we may conclude that the viscoelastic time constants in *Col5a1*^+/−^ corneas are prolonged in short-term and reduced in long-term deformation. In other words, a sudden impact is less damped and thus may lead to rupture. In contrast, on long term steady loads (such as the IOP) more relaxation and thus thinner corneas can be expected. When examining the Prony series fit, the largest difference is observed in the third term (G_3_, τ_3_), which indeed reflects a reduction of the long-term time constant in *Col5a1*^+/−^ corneas. From a biological point of view, thinner corneas could be a consequence of a lower number of collagen fibrils due to the reduced collagen V content^2^.

Overall, the results of the two distinct measurement approaches agree in that corneal E-modulus is increased in *Col5a1*^+/−^ corneas at a measurement time of 10–20 s. We may expect that for even shorter test durations the difference in E-modulus between the two groups would be even higher.

Our SHG microscopy findings demonstrate the importance of collagen V in regulating the stromal hierarchical organization of the cornea. Segev et al.^2^ showed that collagen fibril diameters were increased, but fibril density was decreased in the *Col5a1*^+/−^ corneas by using quantification of fibrils size and spacing using transmission electron microscopy. These alterations in collagen fibril morphology and structure could explain the differences in forward scattered and backward scattered signaling noted in the *Col5a1*^+/−^ corneas. Increased forward scattered and backward scattered signals were present in *Col5a1*^+/−^ corneas. We performed signal intensity measurements (or quantification) of the SHG signal obtained from the WT versus *Col5a1*^+/−^ mice which showed higher signaling in the *Col5a1*^+/−^ mice. However, intensity measruments are hard to interpret and we are currently working on experiments to evaluate if increased collagen content correlates with increased SHG forward scattered and backward scattered signaling.

It is possible that this alteration in fibril morphology and hierarchical organization come with changes in tissue mechanics. Thicker fibrils with increased interfibrillar spacing could hypothetically account for the increased relaxation and brittleness noted in this mouse model.

Besides EDS, the family of ectatic corneal diseases (keratoconus, keratoglobus, pellucid marginal degeneration) shows corneal thinning and altered corneal biomechanics: however, although associations have been described between EDS and keratoglobus^15^, larger series have failed to demonstrate an association with keratoconus. McDermott et al*.* analyzed 72 eyes of 36 patients with genetically-confirmed EDS and found no signs of keratoconus^16^. This might be associated to the fact that *Col5a1*^+*/ −*^ corneas are globally and homogenously deficient in collagen V. In addition, unlike keratoconus corneas, which exhibit localized biomechanical weakening and corneal topographic changes, the higher elastic modulus found here in *Col5a1*^+/−^ corneas support the counterintuitive findings in which collagen V-deficient corneas are able to maintain their physiological shape. In other words, our findings may explain why collagen-deficient thin and abnormal corneas do not develop ectasia.

In OCE measurements, corneal strain values were not different between *Col5a1*^+/−^ and wt, which is not surprising as axial strain alone does not permit a biomechanical interpretation, except the same stress configuration can be ensured. However, this was not the case given that corneas in the *Col5a1*^+/−^ group were 20% thinner than in the wt group. As a consequence, the resulting stress from the 3-mmHg pressure modulation was higher in *Col5a1*^+/−^ corneas. It might be considered a limitation of this study, that the stress level had not been adjusted according to the expected thickness difference. However, as the natural loading condition of the cornea is the intraocular pressure, which is supposedly similar in EDS patients and healthy persons, the application of the same pressure (or force) loading as done in the current study, permits a more physiologically relevant interpretation of the results.

In conclusion, a reduced expression of *Col5a1*^+/−^ in the cornea seems to predominantly affect the viscoelastic properties of the tissue. The results presented here support and rationalize the notion than thin corneas with altered extracellular matrix composition maintain a normal corneal shape during homeostasis. Further studies are needed to evaluate if long-term stress on an abnormal matrix worsen alterations in corneal thickness and facilitate tissue protrusion.

## Methods

### Animals

*A* Col5a1-haploinsufficient mouse model of classic EDS was used in this study^17^. A total of 14 mice heterozygous for collagen V (*Col5a1*^+/−^) and 14 mice of their wild-type littermates (wt) were used for biomechanical analyses. All experiments were conducted ex vivo in enucleated eyes. Each eye underwent first a non-invasive measurement with optical coherence elastography in the intact eye globe, and subsequently destructive 2D extensometry testing in the dissected corneal button. The two techniques applied for mechanical characterization rely on different assumptions and differ in the sensitivity to record deformation and thus assess different locations of the stress–strain curve. For this reason, the combined analysis permits a comprehensive evaluation of tissue properties and allows the comparison of invasive and non-invasive mechanical characterization techniques. OCT measurements are most sensitive to (small) axial displacements and thus strain values were only accessible in vertical direction, i.e. assessing compression. In contrast, extensometry test are most sensitive to (larger) lateral elongation of the corneal tissue, i.e. assessing stretching. Twelve additional eyes were included for second harmonic generation imaging. All animals used were male adults around 60 days old. Mice were housed and treated in accordance with NIH's Guide for the Care and Use of Laboratory Animals. The experiments were conducted in agreement with the ARVO Statement Regarding the Use of Animals in Ophthalmic and Vision Research. All methods were carried out in accordance with relevant guidelines and regulations. All experimental protocols were approved by the institutional animal care and use committee of the University of South Florida.

### Optical coherence elastography (OCE)

Previously, we developed a non-invasive imaging approach to assess corneal stiffness by the use of ambient pressure modulation. This approach was demonstrated to retrieve different irradiation patterns used for CXL treatment in rat eyes^18^. In here, we applied the same technique^18^. Briefly, OCE measurements were conducted with a custom-built spectral domain OCT system (λ = 878 nm, Δλ = 62.5 nm) while the entire eyeball was subjected to ambient pressure modulation. The structural imaging resolution of the spectral domain OCT system was ~ 3.96 μm axially (in tissue) and 12.5 μm laterally; the strain imaging resolution 26 μm axially and 112 μm laterally. Briefly, the eye was placed within a sealed pressure chamber that was connected to a 1 ml syringe and a U-shaped water column. A reference B-scan consisting of 1000 A-scans recorded at a speed of 10 kHz was captured at normal atmospheric pressure (1013 hPa). Subsequently, the pressure within the chamber was reduced by 3 mmHg and a second, deformed B-scan was recorded with the same settings. Phase difference analysis was performed on the two B-scans and converted into induced axial strain Δε_oct_. For this purpose, the complex cross-correlation of the raw OCT signal of the reference and deformed B-scan was computed by

1 $$R={B}_{\mathrm{ref}}\left(z,x\right) \cdot {B}_{\mathrm{def}}^{*}\left(z,x\right),$$

where *B*_ref_ and *B*_def_ respectively represent the reference and deformed cross-sectional B-scan with *z* indicating depth and *x* indicating lateral distance. The axial strain induced between two subsequent B-scans can than be computed from the phase difference according to

2 $${\Delta \varepsilon}_{oct}=\frac{\lambda \cdot {\angle}\left({R}_{\mathrm{s}}(z,x)\cdot {R}_{\mathrm{s}}^{*}(z+1,x)\right)}{4\pi \cdot n\cdot {\mathrm{asu}}},$$

where asu = 4.48 μm represents the axial sampling unit and n = 1.375 the refractive index. Strain values were averaged across the whole thickness and central zone of 0.5 mm diameter. Central corneal thickness (*cct*) was extracted from the OCT scan corneal stress σ_oct_ resulting from the pressure modulation *Δp* was determined with Laplace’s equation^19^:

3 $${\Delta \sigma}_{oct}=\frac{\Delta p\cdot R}{2\cdot cct}$$

where *R* = 1.5 mm is the radius of the cornea. Finally, the E-modulus *E*_*oct*_ was estimated from

4 $${E}_{oct}=\frac{{\Delta \sigma}_{oct}}{{\Delta \epsilon}_{oct}}$$

### 2D extensometry

Our group has recently established a novel method to perform 2D stress–strain extensometry measurements in murine corneas. Using this technique, we could previously demonstrate that corneal cross-linking (CXL) significantly increases corneal stiffness in mice^20^. Extensometry measurements were conducted in corneas circumferentially excised near the limbus. Corneal buttons were fixed within a 2D holder with a central opening of 1.6 mm diameter, as described earlier^20^. A drop of PBS was applied on top and bottom of the opening to prevent dehydration during testing. A hemispherical indenter (1 mm diameter) was then used to apply the test force homogenously over the posterior corneal surface, similar to the IOP. The biomechanical analysis was conducted in three steps: (1) pre-conditioning cycle with three repetitions of stress–strain testing with a load between 0.04 and 0.4 N; (2) stress-relaxation testing during 120 s during the application of an initial load of 0.4 N; and (3) rupture test with an increasing load from 0.4 to 4.0 N. For each step, the tensile corneal stress (i.e. similar to hoop stress) induced by the test load F was determined from a geometrical estimation based on thin shell theory that previously^20^ has been described in more detail:

5 $${\sigma}_{ext}=\frac{F}{2\pi \cdot R\cdot cct}$$

where *cct* is the central corneal thickness determined from OCT measurements. The corresponding 2-dimensional tensile strain was computed from the axial travel distance *Δl* of the indenter following the approach outlined by Hammer et al.^20^:

6 $${\varepsilon}_{ext}=\frac{{\Delta l}^{2}+{R}^{2}}{2\Delta l\cdot R}\cdot {\mathrm{sin}}\left(\frac{2\Delta l\cdot R}{{\Delta l}^{2}+{R}^{2}}\right)-1$$

This assumption holds true, as long as the corneal sample is mounted flat and the induced deformation is in the order of the anterior chamber depth in the mouse eye, which was the case even in rupture tests (25% of corneal tensile strain correspond to an axial travel distance ~ 100 μm). Finally, the E-modulus *E*_*ext*_ was determined from the mean slope in the stress–strain diagram between 10 and 15% of strain. To characterize viscoelastic material properties, a 3-term Prony series was used to describe the stress relaxation curves:

7 $$E={E}_{\infty}+\sum_{i=1}^{N}{E}_{i}\cdot {e}^{-\frac{t}{{\tau}_{i}}},$$

where ${E}_{\infty}$ represents the long-term E-modulus (at full relaxation), *E*_i_ the short-term E-moduli, ${\tau}_{i}$ the corresponding relaxation time constants and N = 3 the number of Prony terms. During stress relaxation strain is kept constant (${\varepsilon}_{ext}=const$), such that after applying the relation $=\frac{{\sigma}_{ext}}{{\varepsilon}_{ext}}$, Eq. (7) can be expressed in terms of stress. In order to retrieve ${E}_{\infty}, {E}_{1} \; to \; { E}_{3} \; and \; {\tau}_{1} \; to \; {\tau}_{3}$, the experimentally measured force *F* was converted to stress according to Eq. (5) and then fitted to Eq. (7) by means of least-squares minimization.

### Second harmonic generation microscopy of corneal stroma

Enucleated eyes were immediately placed in Optisol media on a custom-made glass chamber and imaged (within 5 min) without any tissue manipulation or additional dissection for cross-section images of the cornea. Some corneas were dissected from the globe and placed as a flat mount for en face imaging. Corneas dissected from the globe were immersed in calcein red-orage (ThermoFischer Scientific, Waltham, MA, USA) diluted in optisol medium to allow penetration of the dye to the stroma for 5 min and allow visualization of keratocytes embedded within the stroma. Corneas were imaged using an Olympus MPE-RS microscope using a 25× (0.95 NA) water-immersion objective (Olympus). Two-photon SHG signals were generated using a mode-locked titanium:sapphire laser at 960 nm. The SHG forward-scattered signals passing through the corneal sections were collected using a 0.8 NA condenser lens with a narrow band-pass filter (465–485 nm). Backward-scattered SHG signals were detected with a band pass filter (460–500 nm). All samples were scanned using a 2 μm z-axis step size from the back to the front of the section.

### Statistical analysis

Data distribution was tested for normality with the Shapiro–Wilk test. If normally distributed, parameters were compared with a two-tailed independent student’s t-test, otherwise with the independent samples Mann–Whitney U test. P-values of less than 0.05 were considered statistically significant.

## Data Availability

All data generated or analyzed during this study are included in this published article.
